# Inhaled aerosolised recombinant human activated protein C ameliorates endotoxin-induced lung injury in anaesthetised sheep

**DOI:** 10.1186/cc7777

**Published:** 2009-04-08

**Authors:** Kristine Waerhaug, Vsevolod V Kuzkov, Vladimir N Kuklin, Rica Mortensen, Kåre C Nordhus, Mikhail Y Kirov, Lars J Bjertnaes

**Affiliations:** 1 Department of Anesthesiology, Institute of Clinical Medicine, Faculty of Medicine, University of Tromsø, N-9037 Tromsø, Norway; 2 Department of Radiology, University Hospital of North Norway, Postboks 100, N-9038 Tromsø, Norway; 3 Department of Anesthesiology, Northern State Medical University, Troitzky Avenue 51, 163000 Arkhangelsk, Russian Federation

## Abstract

**Introduction:**

We recently demonstrated that intravenously infused recombinant human activated protein C (APC) attenuates ovine lipopolysaccharide (LPS)-induced lung injury. In this study, our aim was to find out whether treatment with inhaled aerosolised APC (inhAPC) prevents formation of increased lung densities and oedema and derangement of oxygenation during exposure to LPS.

**Methods:**

Sheep were anaesthetised during placement of intravascular introducers. After one to four days of recovery from instrumentation, the animals were re-anaesthetised, endotracheally intubated and mechanically ventilated throughout a six-hour experiment where the sheep underwent quantitative lung computed tomography. Sheep were randomly assigned to one of three groups: a sham-operated group (n = 8) receiving inhaled aerosolised saline from two hours after the start of the experiment; a LPS group (n = 8) receiving an intravenous infusion of LPS 20 ng/kg per hour and, after two hours, inhaled aerosolised saline over the next four hours; a LPS+inhAPC group (n = 8) receiving an intravenous infusion of LPS 20 ng/kg per hour and, after two hours, aerosolised APC 48 μg/kg per hour inhaled throughout the experiment. Data were analysed with analysis of variance; *P *less than 0.05 was regarded as significant.

**Results:**

An infusion of LPS was associated with a reduction of well-aerated lung volume and a rapid fall in arterial oxygenation that were both significantly antagonised by inhaled APC. Pulmonary vascular pressures and extravascular lung water index increased significantly during exposure to LPS, but inhaled APC had no effect on these changes.

**Conclusions:**

Inhalation of aerosolised APC attenuates LPS-induced lung injury in sheep by preventing a decline in the volume of aerated lung tissue and improving oxygenation.

## Introduction

Acute lung injury (ALI) often accompanying sepsis as the first organ failure [1]. When exposed to microbial lipopolysaccharide (LPS), the vasculature responds by activating both the coagulation and inflammation systems. Products released from these systems act on the lung's circulation and its small airways, resulting in vasoconstriction and bronchoconstriction, neutrophil infiltration, vascular leaks, depletion of surfactant, generation of hyaline membranes and closure of small airways [2,3].

Enhanced extravascular lung water (EVLW) content and deteriorated oxygenation are clinical hallmarks of ALI [4]. Recently, investigators noticed a close correlation between lung oedema, determined by thermal dye dilution, and changes in lung volumes, determined with quantitative tomography in patients with acute respiratory distress syndrome (ARDS) [5]. In mechanically ventilated sheep subjected to saline washout-induced lung injury, researchers noticed an increase in extravascular lung water, which was antagonised by positive end-expiratory pressure (PEEP) [6]. The decrease in EVLW also correlated closely with the PEEP-induced fall in non-aerated lung volume and the decline in the transpulmonary shunt [7]. Thus, both EVLW and lung computed tomography (CT) might be useful tools for the assessment of changes in ALI subsequent to therapeutic interventions.

Activated protein C (APC) counteracts coagulation, promotes fibrinolysis [8] and inhibits leucocyte infiltration and, subsequently, the release of pro-inflammatory cytokines [9,10]. We recently reported that intravenously administered recombinant human APC attenuates LPS-induced and oleic acid-induced lung injury in sheep by reducing lung oedema and improving oxygenation [11,12]. Here, we hypothesise that APC reduces LPS-induced lung injury after it is nebulised and inhaled as an aerosol. Thus, our aim was to find out if inhaled APC preserves aeration of lung volumes, as assessed by quantitative CT, prevents the appearance of lung oedema and the derangement of oxygenation in an ovine model of LPS-induced lung injury.

## Materials and methods

The experiment was approved by the Norwegian Experimental Animal Board and performed in accordance with the Helsinki Convention for Use and Care of Animals.

Sheep were instrumented under sterile conditions during general anaesthesia induced with thiopental sodium (Abbott, North Chicago, IL, USA) 15 to 20 mg/kg intravenously for endotracheal intubation and maintained with isoflurane 1.2 to 1.5% (Abbott Scandinavia AB, Kista, Sweden). An 8.5 Fr introducer (CC-350B; Baxter, Irvine, CA, USA) was inserted transcutaneously into the left external jugular vein and a 5 Fr introducer was inserted into the ipsilateral carotid artery (CP-07511-P; Arrow international, Reading, PA, USA). Postoperatively, sheep were allowed to recover from instrumentation for one to four days with free access to food and water while receiving buphrenorphine (Temgesic, Schering-Plough Corporation, Kenilworth, NJ, USA) 0.6 mg subcutaneously twice daily. Antibiotic prophylaxis was performed with enrofloxacin (Bayer Health Care, Monheim, Germany). Introducers and catheters were flushed twice daily with heparin (Heparin, LEO Pharma AS, Ballerup, Denmark) 5 mIU dissolved in 5 mL of saline.

On the day of the experiment, the sheep were anaesthetised with thiopental dosed as above for endotracheal intubation, and subsequently sedated with fentanyl (Leptanal; Janssen Pharmaceuticals, Beerse, Belgium) 0.05 mg/kg per hour and midazolam (Dormicum; Roche, Basel, Switzerland) 1 mg/kg per hour. Additional intravenous bolus doses of fentanyl and midazolam were administered intermittently as necessary with no use of muscle relaxants. Positive pressure ventilation was employed using a Servo 900 C respirator (Siemens-Elema, Solna, Sweden) with tidal volume set at a constant 6 ml/kg, fraction of inspired oxygen (FiO_2_) of 0.21, respiratory rate of 20 breaths/minute and zero PEEP. A Swan Ganz catheter (131HF7; Baxter Healthcare, Irvine, CA, USA) was inserted into the pulmonary artery via the introducer in the external jugular vein, and a thermodilution catheter (PV2014L16; Pulsion Medical Systems, Munich, Germany) was inserted into the aorta through the introducer in the carotid artery.

### Experimental protocol

Sheep were randomly assigned to either a sham-operated group (Sham group, n = 8) or one of two LPS groups receiving an intravenous infusion of *Eschericia coli *lipopolysaccharide O26:B6 (Sigma Chemical, St. Louis, MO, USA) 20 ng/kg per minute for six hours. One group received LPS only (LPS group, n = 8) and one group additionally received APC (Xigris^®^, Eli Lilly & Co, Indianapolis, IN, USA) 48 μg/kg per hour, which was dissolved in 5 mL of isotonic saline, nebulised with an ultrasound nebuliser (Servo Ultra 145, Siemens-Elema, Solna, Sweden) and inhaled as an aerosol from two to six hours after the start of LPS treatment (LPS+inhAPC-group, n = 8). Correspondingly, the Sham and the LPS groups inhaled 5 mL per hour of aerosolised solvent. Aerosolised APC or solvent was administered in the inspiratory arm of the breathing system. Sheep in the Sham and LPS groups received 3 and 5 mL/kg per hour, respectively, of a continuous intravenous infusion of Ringer's acetate.

Blood samples were drawn from the systemic (a) and pulmonary artery (v) lines every second hour and analysed for blood gases and haemoglobin (Rapid 860; Chiron Diagnostics Corporation, East Walpole, MA, USA). Body temperature (Temp) was taken from the thermodilution catheter in the aorta. Venous admixture (Qs/Qt) and alveolo-arterial oxygen tension difference (AaPO_2_) were determined according to standard formulas.

Haemodynamics were monitored every second hour employing a Swan Ganz-catheter in the pulmonary artery and a thermodilution catheter in the aorta connected to the PiCCO plus monitor (PiCCO plus; Pulsion Medical Systems, Munich, Germany). The single transpulmonary thermodilution technique was used to determine the cardiac index (CI) and derived volumetric data. The recorded variables included pulmonary artery pressure (PAP), pulmonary arterial occlusion pressure (PAOP), pulmonary micro-occlusion pressure (Pmo), pulmonary vascular resistance index (PVRI), heart rate (HR), CI, systemic arterial blood pressure (MAP), systemic vascular resistance index (SVRI), right atrial pressure (RAP), left ventricular stroke work index (LVSWI), right ventricular stroke work index (RVSWI) and extravascular lung water index (EVLWI). Derived volumetric variables were calculated as described previously by our group [13].

After six hours, a spiral lung CT (Somatom sensation 16) with slice thickness (voxels) of 5 mm was carried out, while clamping the tube for 15 seconds at functional residual capacity (FRC) and at end-inspiration, respectively. Delineation of the lung contour with exclusion of vessels and bronchi was manually performed by a radiologist blinded to which group the sheep belonged to. Acquisition and analysis of CT data was performed using the Pulmo CT program (Siemens, Munich, Germany). Density of the lungs was determined in Houndsfield Units (HU). A CT-number equal to 0 HU characterises a voxel whose density is equal to that of water and a CT number of -1000 HU characterises a voxel whose density is equal to that of air.

The CT number is linearly correlated with density [14], so quantitative analysis of weight and gas content of lung tissue could be performed [15]. Regions of pre-defined densities were identified, and their volumes were calculated automatically by the program. Volumes containing lung densities ranging from -1000 to -500 HU were defined as 'well aerated' lung, and correspondingly, 'poorly aerated' lung volumes ranged from -500 to -100 HU and 'non-aerated' lung volumes ranged from -100 to +100 HU. Tissue volume index was calculated according to the formula:



where TVI_CT _= CT-derived relative lung tissue volume index (mL/kg); V_WA _= volume of well aerated lung (mL/kg); V_PA _= volume of poorly aerated lung (mL/kg); V_NA _= volume of non-aerated lung (mL/kg).

The volume of gas in each lung was calculated as:



The animals were euthanased with an intravenous injection of pentobarbital (Pentobarbital NAF, Ås Production Lab, Ås, Norway) 100 mg/kg followed by a bolus injection of potassium chloride (KCl NAF, Ås Production Lab, Ås, Norway) 300 mmol.

### Statistical analysis

Data were analysed using a two-factor analysis of variance for repeated measurements (SPSS 14.0 for Windows, LEAD Technologies, Chicago, IL, USA) and expressed as mean ± standard error of the mean (SEM). To evaluate differences within groups over time and interaction between time and group, we employed a test of contrasts. Greenhouse-Geisser epsilon factor adjusted probability levels were used when Mauchly's test was significant. To test for differences in aeration estimated by CT we applied Student's t-test or Mann-Whitney U-test when appropriate. We regarded *P *less than 0.05 as statistically significant.

## Results

**Figure 1:**
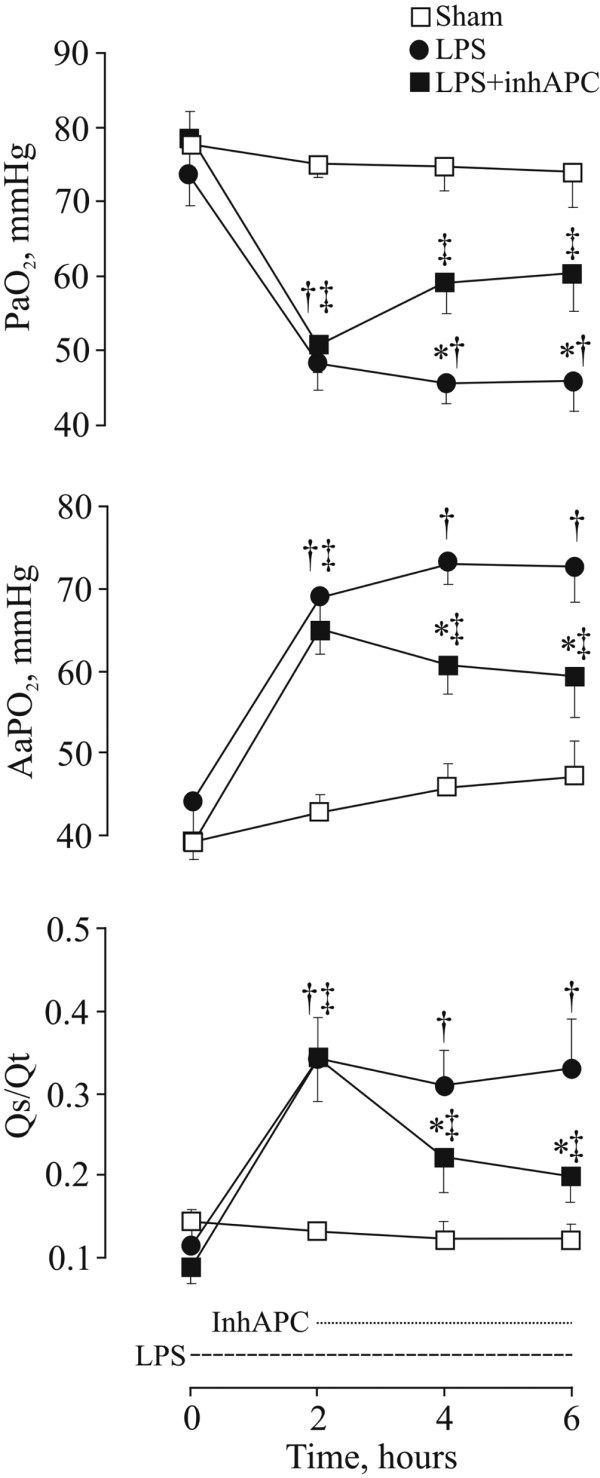
Changes in oxygenation variables in anaesthetised mechanically ventilated sheep. Data presented as the mean ± standard error of the mean. Sham = sham-operated group (n = 8); LPS = lipopolysaccharide group (n = 8); LPS+inhAPC = LPS group treated with inhaled recombinant human activated protein C (n = 8); PaO_2 _= arterial oxygen partial pressure; AaPO_2 _= alveolar–arterial oxygen tension difference; Qs/Qt = venous admixture. *P < 0.05 between LPS and LPS+inhAPC-groups; †*P *< 0.05 from t = 0 hours in LPS-group; ‡*P *< 0.05 from t = 0 hours in LPS+inhAPC-group.

All sheep survived the experiments. There were no significant differences between the groups at baseline. As shown in Figure 1, infusion of LPS rapidly deteriorated oxygenation as evidenced by a decrease in partial pressure of oxygen in arterial blood (PaO_2_; *P *< 0.001) in parallel with rises in AaPO_2 _and Qs/Qt (*P *< 0.001 for both variables). InhAPC antagonised the decline in oxygenation with significant intergroup differences in PaO_2 _(*P *= 0.009), AaPO_2 _(*P *= 0.002) and Qs/Qt (*P *= 0.025). Table 1 displays that oxygen saturation (SaO_2_) and mixed venous oxygen saturation (SvO_2_) both improved significantly on inhalation of APC, but without significant intergroup differences compared with LPS alone. Blood temperature increased in all groups, including sham-operated sheep (*P *= 0.02). The latter group did not reach the levels of animals exposed to LPS alone (*P *< 0.001). An early increase in haemoglobin concentration was observed in both LPS groups (*P *< 0.001). Inhaled APC displayed no significant effects on the changes in haemoglobin or blood temperature. Except for a slight increase in pH (*P *= 0.002) and a decrease in partial pressure of carbon dioxide in arterial blood (PaCO_2_; *P *= 0.013) after inhaled APC, we noticed no changes in acid-base balance.

**Table 1:** Effects of inhaled activated protein C on lipopolysaccharide-induced changes in oxygen related variables and body temperature.

	0 hour	2 hours	4 hours	6 hours
SaO_2_, %				
Sham	97 ± 1	98 ± 0	98 ± 1	98 ± 1
LPS	97 ± 1	86 ± 3^a^	84 ± 3^a^	84 ± 4^a^
LPS+inhAPC	98 ± 1	86 ± 3^a^	93 ± 2^ab^	94 ± 1^ab^
SvO_2_, %				
Sham	68 ± 2	69 ± 2	68 ± 2	67 ± 2
LPS	64 ± 2	58 ± 4	51 ± 4^a^	54 ± 5^a^
LPS+inhAPC	68 ± 2	58 ± 3^a^	65 ± 3^bc^	64 ± 4^b^
Temp, C°				
Sham	38.1 ± 0.3	38.4 ± 0.4	38.8 ± 0.4^ab^	38.8 ± 0.4^ab^
LPS	38.3 ± 0.3	39.4 ± 0.3^a^	40.2 ± 0.2^ab^	40.4 ± 0.3^ab^
LPS+inhAPC	38.3 ± 0.1	39.4 ± 0.1^a^	40.3 ± 0.1^ab^	40.5 ± 0.1^ab^
Hb, g/dL				
Sham	8 ± 0	9 ± 0	9 ± 0	8 ± 0
LPS	9 ± 1	11 ± 1^a^	11 ± 1^a^	11 ± 1^a^
LPS+inhAPC	9 ± 0	12 ± 0^a^	12 ± 0^a^	11 ± 0^a^
pH				
Sham	7.53 ± 0.01	7.54 ± 0.01	7.56 ± 0.01	7.53 ± 0.01
LPS	7.52 ± 0.01	7.51 ± 0.02	7.51 ± 0.02	7.52 ± 0.02
LPS+inhAPC	7.51 ± 0.01	7.48 ± 0.02	7.53 ± 0.02^bc^	7.54 ± 0.01^ab^
PaCO_2_				
Sham	37 ± 2	36 ± 2	35 ± 1	34 ± 2
LPS	40 ± 2	41 ± 2	41 ± 2	39 ± 2
LPS+APC	40 ± 1	42 ± 3	37 ± 2^b^	37 ± 1^b^

Data presented as the mean ± standard error of the mean.

Hb = haemoglobin; LPS = sheep receiving infusion of lipopolysaccharide (n = 8); LPS+inhAPC = sheep receiving LPS and inhaled nebulised recombinant human activated protein C (n = 8); PaCO_2 _= arterial carbon dioxide partial pressure; pH = arterial pH;. SaO_2 _= arterial oxygen saturation; Sham = sham operated sheep (n = 8); SvO_2 _= central venous oxygen saturation; Temp = body temperature.

^a^*P *< 0.05 from t = 0 hours; ^b^*P *< 0.05 from t = 2 hours; ^c^*P *< 0.05 between LPS and LPS+inhAPC-groups.

**Figure 2:**
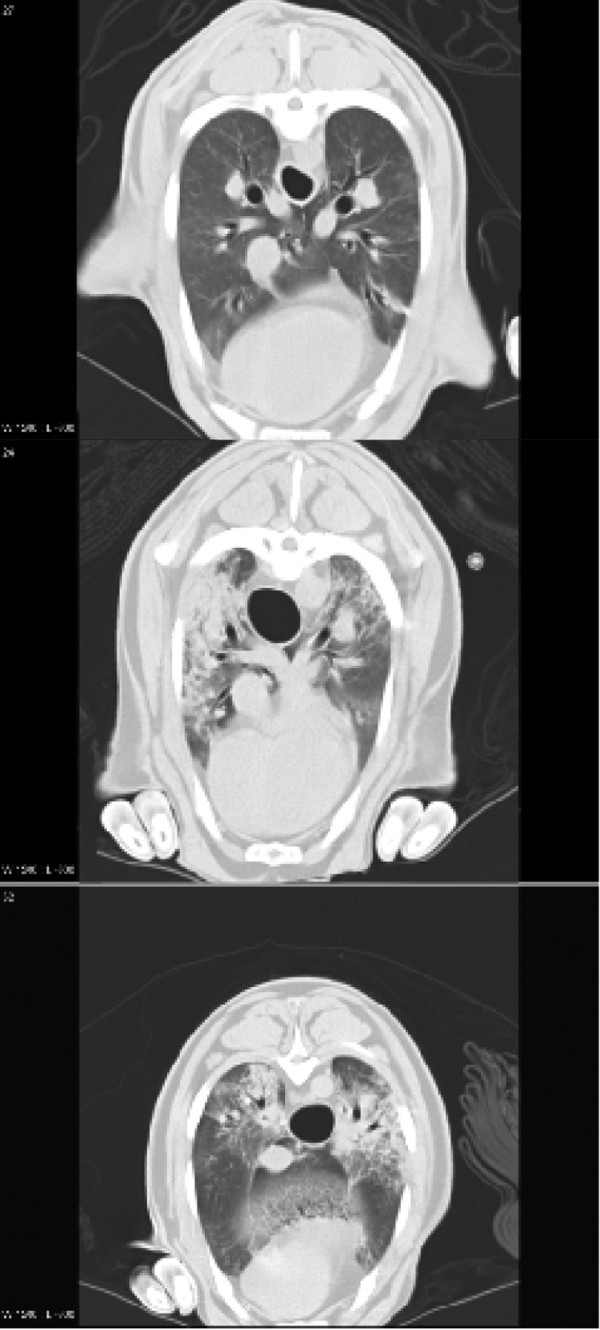
Representative lung computed tomography scans of anaesthetised mechanically ventilated sheep taken at functional residual capacity. Top: sham-operated sheep. Middle: sheep subjected to continuous infusion of lipopolysaccharide (LPS). Bottom: sheep subjected to LPS and treated with aerosolised inhaled recombinant human activated protein C.

**Figure 3:**
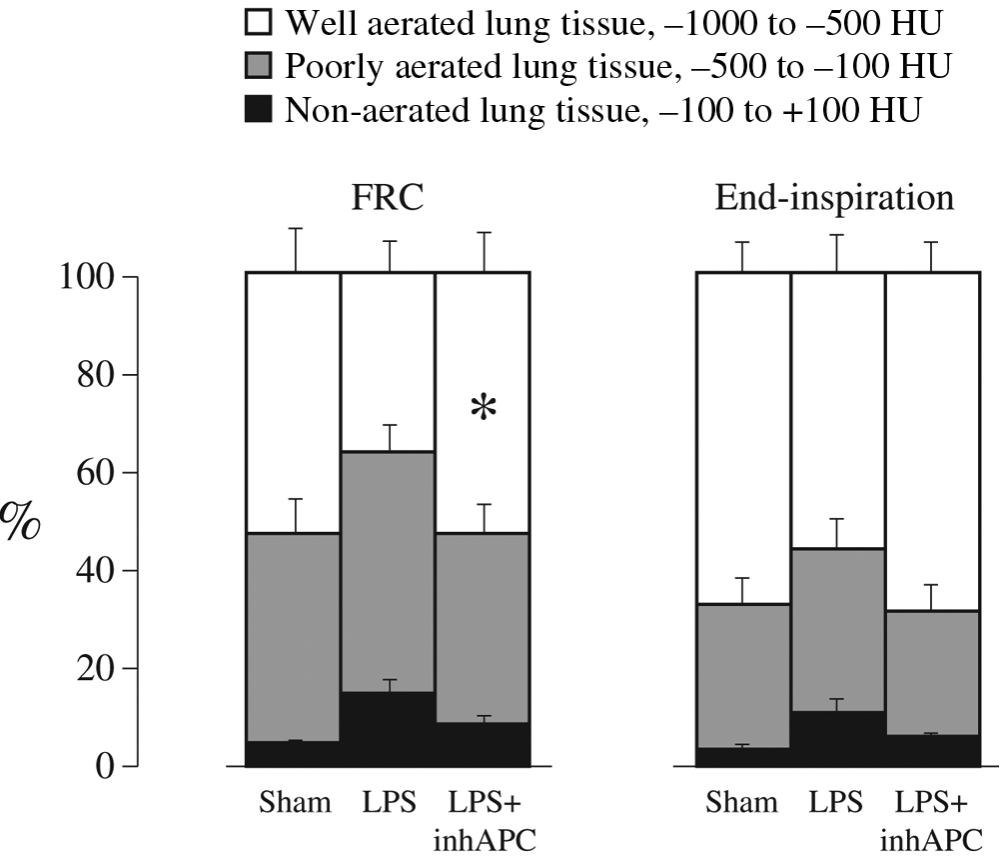
Volumes of well-aerated, poorly aerated and non-aerated lung determined with quantitative computed tomography at functional residual capacity and at end inspiration in anaesthetised mechanically ventilated sheep. Data presented as the mean ± standard error of the mean. FRC = functional residual capacity; LPS = lipopolysaccharide group (n = 8); LPS+inhAPC = LPS group treated with aerosolised inhaled recombinant human activated protein C (n = 8); Sham = sham-operated group (n = 8). * *P *< 0.05 between LPS and LPS+inhAPC groups.

**Figure 4:**
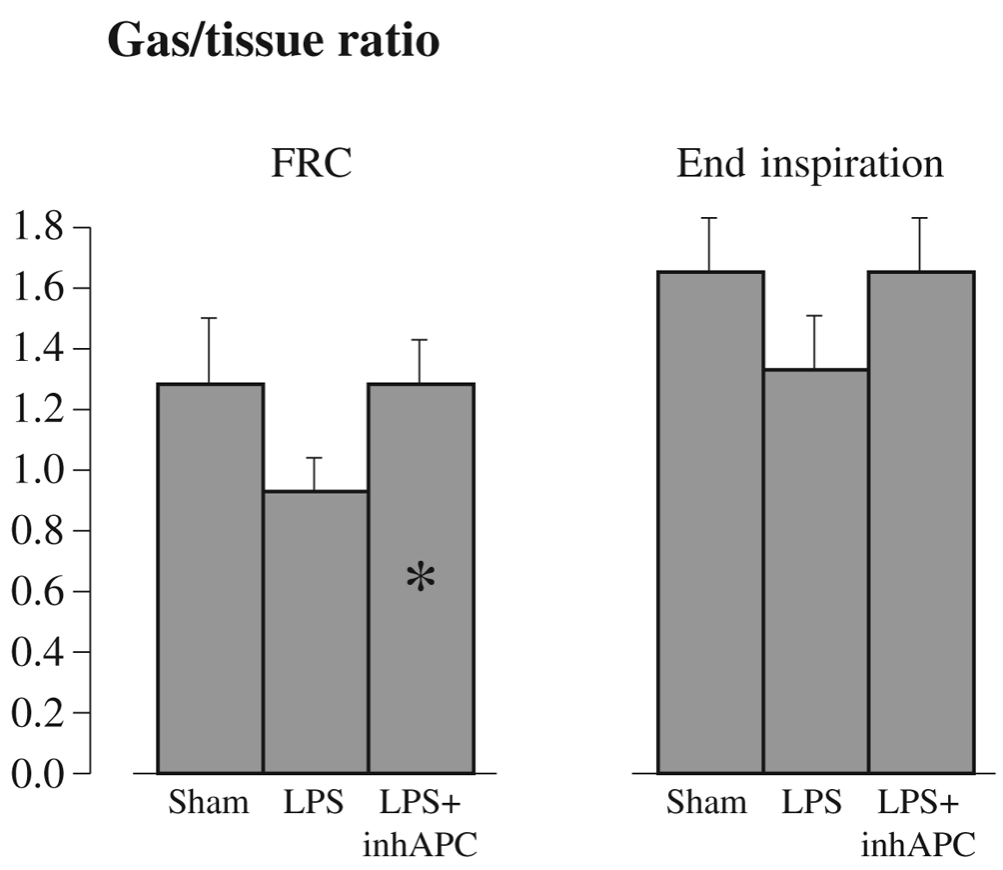
Lung gas/tissue ratio determined with quantitative computed tomography at functional residual capacity and at end inspiration in anaesthetised mechanically ventilated sheep. Data presented as the mean ± standard error of the mean. FRC = functional residual capacity; LPS = lipopolysaccharide group (n = 8); LPS+inhAPC = LPS group treated with aerosolised inhaled recombinant human activated protein C (n = 8); Sham = sham-operated group (n = 8). * *P *< 0.05 between LPS and LPS+inhAPC groups.

Figure 2 shows representative CT scans taken at FRC of an animal from the sham group (top), one from the LPS (middle) group and one from the LPS+inhAPC (bottom) group. As depicted in Figure 3, at FRC the LPS+inhAPC group presented with a 50% higher volume of well-aerated lung tissue as compared with animals exposed to LPS alone (*P *= 0.03). However, when examined at end-inspiration, the difference was no longer significant, with an average of 66% well-aerated lung tissue volume in the LPS+inhAPC group compared with 53% with LPS alone (*P *= 0.06). Correspondingly, when examined at FRC, the gas/tissue ratio was also higher in the LPS+inhAPC group as compared with sheep exposed to LPS alone (Figure 4; *P *= 0.03), but the intergroup difference was not present at end inspiration (*P *= 0.09). Total lung gas and tissue volumes (data not presented) displayed no significant intergroup differences at FRC or end inspiration.

PAP, PAOP, Pmo, PVRI and HR all increased significantly with infusion of LPS and remained elevated throughout the experiment (Table 2). After LPS, CI increased transiently without significant differences within or between the groups. MAP tended to decrease during exposure to LPS (*P *= 0.06) and SVRI declined gradually (*P *= 0.02) in concert with almost a doubling of RAP (*P *< 0.001). Moreover, LVSWI decreased (*P *= 0.01) while RVSWI and EVLWI increased slightly during infusion of LPS (*P *= 0.006 and 0.02, respectively). However, inhaled APC had no significant effects on any of the LPS-induced changes in these haemodynamic and volumetric variables.

**Table 2:** Effects of inhaled activated protein C on lipopolysaccharide-induced changes in systemic and pulmonary haemodynamic variables in sheep.

	0 hour	1 hour	2 hours	4 hours	6 hours
PAP, mmHg					
Sham	15 ± 1	16 ± 1	16 ± 1	17 ± 1	17 ± 1
LPS	18 ± 0	46 ± 2^a^	32 ± 2^a^	35 ± 2^a^	33 ± 2^a^
LPS+inhAPC	17 ± 0	47 ± 2^a^	30 ± 1^a^	31 ± 1^a^	29 ± 1^a^
PAOP, mmHg					
Sham	7 ± 1	7 ± 1	8 ± 1	8 ± 1	7 ± 1
LPS	9 ± 1	13 ± 1^a^	12 ± 1^a^	14 ± 1^ab^	14 ± 1^ab^
LPS+inhAPC	9 ± 1	14 ± 1^a^	11 ± 1^a^	13 ± 1^a^	12 ± 1^a^
Pmo, mmHg					
Sham	6 ± 1		7 ± 1	7 ± 1	7 ± 1
LPS	9 ± 0		13 ± 1^a^	14 ± 1^a^	15 ± 1^a^
LPS+inhAPC	9 ± 1		13 ± 1	12 ± 1^a^	12 ± 1
PVRI, dyne·sec/cm^5^/m^2^					
Sham	144 ± 18	151 ± 25	156 ± 35	173 ± 26	191 ± 18
LPS	179 ± 12	665 ± 107^a^	353 ± 49^a^	433 ± 64^a^	339 ± 56^a^
LPS+inhAPC	165 ± 16	563 ± 63^a^	351 ± 31^a^	379 ± 37^a^	350 ± 33^a^
HR, beats/min					
Sham	102 ± 6	111 ± 6	111 ± 6	107 ± 7	100 ± 8
LPS	100 ± 9	142 ± 15^a^	154 ± 11^a^	131 ± 10^ab^	140 ± 5^a^
LPS+inhAPC	93 ± 10	160 ± 16^a^	160 ± 15^a^	121 ± 4^ab^	128 ± 6^ab^
CI, L/min/m^2^					
Sham	4.5 ± 0.4	4.6 ± 0.4	4.1 ± 0.2	4.2 ± 0.3	4.3 ± 0.3
LPS	4.0 ± 0.3	5.0 ± 0.9	4.8 ± 0.3	4.6 ± 0.6	4.6 ± 0.4
LPS+inhAPC	4.2 ± 0.3	5.3 ± 0.7	4.7 ± 0.3	4.1 ± 0.2	4.2 ± 0.4
MAP, mmHg					
Sham	112 ± 5	118 ± 6	118 ± 6	116 ± 5	112 ± 6
LPS	108 ± 6	102 ± 6	96 ± 6	103 ± 6	93 ± 5
LPS+inhAPC	110 ± 6	110 ± 4	102 ± 5	111 ± 8	108 ± 9
SVRI, dyne·sec/cm^5^/m^2^					
Sham	2068 ± 230	2146 ± 234	2261 ± 179	2251 ± 207	2139 ± 226
LPS	2159 ± 179	2003 ± 466	1577 ± 146	1824 ± 243	1499 ± 154^a^
LPS+inhAPC	2151 ± 174	1849 ± 245	1839 ± 179	2188 ± 249	2130 ± 290
RAP, mmHg					
Sham	2 ± 1	3 ± 1	3 ± 1	3 ± 1	3 ± 1
LPS	5 ± 1	5 ± 1	5 ± 1	9 ± 1^ab^	10 ± 1^ab^
LPS+inhAPC	3 ± 0	3 ± 1	3 ± 1	6 ± 1^ab^	8 ± 1^ab^
LVSWI, g·m/m^2^					
Sham	63 ± 4	72 ± 9	56 ± 4	58 ± 6	60 ± 6
LPS	52 ± 7	42 ± 6	36 ± 5^a^	39 ± 3	33 ± 3^a^
LPS+inhAPC	66 ± 6	42 ± 4	36 ± 4^a^	43 ± 3^a^	41 ± 4^a^
RVSWI, g·m/m^2^					
Sham	7 ± 1	9 ± 2	6 ± 1	8 ± 1	8 ± 2
LPS	7 ± 1	19 ± 3^a^	11 ± 1^a^	11 ± 1^a^	9 ± 1^a^
LPS+inhAPC	9 ± 1	19 ± 2	11 ± 1	12 ± 1	10 ± 1
EVLWI, mL/kg					
Sham	10 ± 1		10 ± 0	10 ± 0	10 ± 0
LPS	10 ± 1		11 ± 1	11 ± 0	12 ± 1^a^
LPS+inhAPC	9 ± 0		11 ± 1^a^	10 ± 1	11 ± 1^a^

Data presented as the mean ± standard error of the mean.

CI = cardiac index; EVLWI = extravascular lung water index; HR = heart rate; LPS = sheep receiving infusion of lipopolysaccharide (n = 8); LPS+inhAPC = sheep receiving LPS and inhaled nebulised recombinant human activated protein C (n = 8); LVSWI = left ventricle stroke work index; MAP = mean systemic arterial pressure; PAP = pulmonary artery pressure; PAOP = pulmonary artery occlusion pressure; Pmo = pulmonary micro occlusion pressure; PVRI = pulmonary vascular resistance index; RAP = right atrial pressure; RVSWI = right ventricle stroke work index;Sham = sham operated sheep (n = 8); SVRI = systemic vascular resistance index.

^a^*P *< 0.05 from t = 0 hours; ^b^*P *< 0.05 from t = 2 hours.

## Discussion

The present study is the first in a larger animal model showing that treatment with inhAPC counteracts LPS-induced ALI by improving gas exchange, and maintaining well-aerated lung volumes and lung gas/tissue ratio, as assessed by quantitative CT.

Our findings confirm the results of investigations showing that APC possesses the ability to modulate various forms of ALI in sheep [11,12,16,17]. Employing an ovine model of endotoxin-induced lung injury, our group noticed that intravenously infused APC, at the same dose as used for treatment of severe sepsis, improves oxygenation and reduces the increments in Pmo and EVLW [11]. However, apart from oxygenation, which reached criteria of ALI in six animals and ARDS in two animals of the LPS-group [18] and improved markedly on treatment with inhaled APC, none of the latter variables changed significantly in the present study.

We interpret the decrease in PaCO_2 _and the parallel rise in pH following inhalation of APC (Table 1) as indications of improved alveolar ventilation because the ventilator settings were kept constant from the beginning to the end of the experiments.

The improvement of oxygenation in parallel with the demonstration of larger volumes of well-aerated lung at FRC indicates that inhaled APC may have prevented the emergence of densities occurring with LPS alone (Figures 2 and 3). Such densities are usually seen in patients with ALI [4,19,20]. In support of these observations, investigators recently reported a patient with severe sepsis-induced ARDS who presented with a resolution of the diffuse infiltrates on the chest x-ray in concert with increased PaO_2_/FiO_2 _ratio after four days of treatment with inhaled APC [21].

The fact that the gas/tissue ratio was higher in APC-treated animals compared with sheep exposed to LPS alone (Figure 4) is most likely a result of more atelectasis in the latter group. The intergroup difference of well-aerated lung volumes vanished at end inspiration probably because the LPS group had more recruitable lung tissue compared with the group treated with inhaled APC. In the LPS group, larger parts of the airways closed during the expiration. Most likely, this closure could be reduced or prevented by the application of PEEP, as shown in patients with ARDS [15]. We believe that the LPS+inhAPC group had more open airways as a result of the treatment. These findings agree with the observations of reduced peak airway pressure and bronchial obstruction after APC in an ovine model of lung injury after smoke inhalation and sepsis [16].

APC appeared to be less effective in dampening LPS-induced lung haemodynamic responses when administered directly into the airways, compared with the intravenous route [11,16,17]. Concerning PAP, PAOP and Pmo, their responses to LPS did not differ significantly from the corresponding responses we observed previously [11]. Although these variables tended to decrease during inhaled APC, we found no differences compared with those observed with LPS alone. However, the present investigation was probably underpowered to unveil these possible effects. Reaching intergroup differences with 80% power at a probability of 5% would require sample sizes of 30 animals or more in each group. The limited effects on pulmonary haemodynamics may be explained by a lower systemic concentration of the active drug, which could be caused by limited absorption of APC from the airways or even a damaging effect of the nebuliser on the APC molecules.

In the present study, EVLWI increased by only 23% with LPS alone (*P *= 0.03). This is a small increase compared with the more than 100% increments we observed in endotoxaemic spontaneously breathing sheep studied awake [11,22-24]. Mechanical ventilation reduces the transmural microvascular pressure, which might explain the decreased lung oedema formation in the present study, but when PEEP is applied this effect may be partly outweighed by a decrease in lung lymph flow [25]. Additionally, we suspect that the batch of *E. coli *LPS, which we used in this and a concurrent investigation [13], was less potent than that employed in previous experiments concerning the induction of lung oedema.

The modest lung oedema could not account for the significant hypoxaemia noticed in this study. The derangement of oxygenation, most likely, resulted from the emerging atelectasis. In atelectatic and poorly ventilated areas of lungs, the pulmonary vasoconstrictor response to hypoxia (HPV) normally acts to redistribute blood flow to better ventilated areas [26]. It is well established that infusion of LPS, or sepsis, hampers HPV by inducing endogenous production of the vasodilator nitric oxide [27,28]. In sepsis, HPV recovers after inhibition of inducible nitric oxide synthase [28]. Sheep subjected to combined smoke inhalation and sepsis, presented with reduced levels of lung tissue of the nitric oxide metabolite tri-nitrotyrosine after intravenously administered APC [16]. APC also prevented endotoxin-induced hypotension in rats by inhibiting excessive nitric oxide production [29]. Thus, we speculate that preservation of HPV, at least in part, can explain the improvement of oxygenation in animals treated with inhaled APC.

We suggest that the present attenuation of ALI is caused by anticoagulant, anti-inflammatory and profibrinolytic effects of APC, as we have demonstrated previously in endotoxaemic sheep [11]. Consistent with our previous observations, we did not notice any signs of bleeding from the airways, or from any other organ system after inhaled APC. The possibility of any hidden bleeding into the lung interstitium after exposure to inhaled APC is also less likely because such an event would worsen rather than improve oxygenation and the gas/tissue ratio.

The present study has limitations. We did not assess the distribution of inhaled APC within the lungs or its final plasma concentration, thus necessitating further studies. However, visual inspection revealed that a vapour containing the aerosolised solution of APC entered the endotracheal tube during inspiration. Taking into account a molecular weight of more than 50 kD, we assume that the size of the aerosolised particles might have limited their access to the smallest airways. Nevertheless, the clinical effects, particularly on oxygenation and lung aeration, make us believe that some of the active drug reached the distal airways. However, the fact that we observed no significant effects on LPS-induced changes in pulmonary haemodynamics, indicate that systemic absorption was sparse. Although the present findings cannot be immediately extrapolated to humans, they suggest a role for inhAPC, particularly in patients with sepsis-induced ALI at increased risk of bleeding. We recommend that inhaled APC should be subjected to a clinical trial in patients with ALI or ARDS after sepsis.

## Conclusions

The present study demonstrates that treatment of ovine endotoxin-induced lung injury with aerosolised inhaled recombinant human APC improves oxygenation, and maintains aeration of lung tissue, as assessed by CT.

## Key messages

• In anaesthetised mechanically ventilated sheep, treatment with aerosolised inhaled recombinant human APC counteracts the decline in well-aerated lung volumes and the deterioration of oxygenation after endotoxin-induced lung injury.

## Abbreviations

AaPO_2_: alveolar-arterial oxygen tension difference; ALI: acute lung injury; APC: activated protein C; ARDS: acute respiratory distress syndrome; CI: cardiac index; CT: computed tomography; EVLW: extravascular lung water; EVLWI: extravascular lung water index; FiO_2_: fraction of inspired oxygen; FRC: functional residual capacity; HPV: pulmonary vasoconstrictor response to hypoxia; HR: heart rate; inhAPC: inhaled aerosolised recombinant human activated protein C; LPS: lipopolysaccharide; LVSWI: left ventricular stroke work index; MAP: mean systemic arterial pressure; PaCO_2_: partial pressure of carbon dioxide in arterial blood; PaO_2_: partial pressure of oxygen in arterial blood; PAOP: pulmonary arterial occlusion pressure; PAP: pulmonary arterial pressure; PEEP: positive end-expiratory pressure; Pmo: pulmonary capillary micro-occlusion pressure; PVRI: pulmonary vascular resistance index; Qs/Qt: venous admixture; RAP: right atrial pressure; RVSWI: right ventricular stroke work index; SaO_2_: oxygen saturation; SEM: standard error of the mean; SvO_2_: mixed venous oxygen saturation; SVRI: systemic vascular resistance index; TVI_CT_: tissue volume index

## Competing interests

The present study was supported by Helse Nord (Project Number 4001.721.477), the Departments of Anesthesiology, University Hospital of North Norway and Institute of Clinical Medicine, University of Tromsø, Tromsø, Norway. We appreciate the donation of APC (Xigris^®^) provided by Eli Lilly & Co, Indianapolis, IN, USA. MYK is a member of the Advisory Board of Pulsion Medical Systems (München, Germany).

## Authors' contributions

KW participated in the design of the study and in the experiments, analysed the data, prepared figures and tables and partly drafted the manuscript. VNK, VVK and MK participated in the design of the study and in the experimentation. RM and KN performed the computed tomography and analysed the results without knowledge of which experimental group the sheep belonged to. LB participated in the administration and the design of the study, and drafted the manuscript. All authors have read and approved the final manuscript.
